# Fuzzy constraint-based agent negotiation framework for doctor-patient shared decision-making

**DOI:** 10.1186/s12911-022-01963-x

**Published:** 2022-08-13

**Authors:** Kaibiao Lin, Yong Liu, Ping Lu, Yimin Yang, Haiting Fan, Feiping Hong

**Affiliations:** 1 School of Computer and Information Engineering, Xiamen University of Technology, Xiamen, 361024 China; 2 Engineering Research Center of Big Data Application in Private Health Medicine, Fujian Provincial University, Putian, 351100 China; 3 School of Economic and Management, Xiamen University of Technology, Xiamen, 361024 China; 4 Key Laboratory of Ecological Environment and Information Atlas, Fujian Provincial University, Putian, 351100 China; 5 Department of Pediatrics, Xiamen Hospital of Traditional Chinese Medicine, Xiamen, 361024 China; 6 Department of Neonates, Xiamen Humanity Hospital, Xiamen, 361024 China

**Keywords:** Shared decision-making, Agent, Fuzzy constraint, Negotiation, Treatments recommendation

## Abstract

**Background:**

The clinical practice of shared decision-making (SDM) has grown in importance. However, most studies on SDM practice concentrated on providing auxiliary knowledge from the third-party standpoint without consideration for the value preferences of doctors and patients. The essences of these methods are complete and manual negotiation, and the problems of high cost, time consumption, delayed response, and decision fatigue are serious.

**Methods:**

In response to the above limitations, this article proposes a fuzzy constraint-directed agent-based negotiation and recommendation framework for bilateral and multi-issue preference negotiation in SDM (PN-SDM). Its purpose is to provide preference information and intellectualize PN-SDM to promote SDM practice. We modeled PN-SDM problems as distributed fuzzy constraint satisfaction problems and designed the doctor agent and patient agent to negotiate on behalf of the doctor and patient. The negotiation result was then transformed into treatment plans by the recommendation model. The proposed negotiation and recommendation models were introduced in detail by an instance.

**Results:**

The proposed method with different strategies and negotiation pairs achieves good performance in terms of negotiation running time, negotiation rounds, and combined aggregated satisfaction value. Specifically, it can feasibly and effectively complete multiple rounds of PN-SDM in a few seconds and obtain higher satisfaction.

**Conclusion:**

The experimental results indicate that the negotiation model can effectively simulate preference negotiation and relieve the pressure of increasing issues. The recommendation model can assist in decision-making and help to realize SDM. In addition, it can flexibly cope with various negotiation scenarios by using different negotiation strategies (e.g., collaborative, win–win, and competitive).

## Introduction

Shared decision-making (SDM) is gradually being advocated as an ideal treatment decision-making method in medical practice. It refers to doctor and patient reaching agreements on how to diagnose, treat and administer drugs and whether to carry out special examinations or operations after full consultation [1]. It aims to help patient play an active role in the decision-making process and pay attention to their health, which is the ultimate goal of patient-centered care [2]. SDM was first proposed in 1972 [3], and that expected to satisfy the demands of medical ethics, clinical practice, and disease management [4]. Furthermore, it is designed to relieve the growing tension between doctor and patient [5] and improve patient compliance [6] and treatment effects [7, 8].

However, the clinical application of SDM faces a variety of challenges [9, 10]. For example, the lack of communication awareness and skills of doctors or patients, the limited time of doctors, and the lack of patients’ medical knowledge. To better implement SDM, numerous experts and scholars have explored SDM in detail and presented many feasible solutions. For instance, the development of patient decision aid tools [11, 12], skills training for SDM [13], and continuing medical education [14]. Although these measures can solve the above problems to some extent, the essence of SDM is still complete manual negotiation. Problems such as high cost, time consumption, delayed response, and decision fatigue still exists and are serious. Psychological studies show that individuals may have decision fatigue when making decisions. In particular, this phenomenon is more likely to occur in a clinical environment that faces a large number of medical decisions every day [15]. Decision fatigue not only reduces individual willpower, self-discipline, and self-control but also leads to low decision-making quality [16].

Some scholars have also developed clinical decision-support systems [17–19]. It aims to establish human–machine interactive medical systems through data or models to assist doctors and patients in clinical decisions [20]. These systems include knowledge base systems such as Quick Medical Reference [21], UpToDate [22], and BMJ Best Practice [23] and non-knowledge base systems such as Archimedes Model [24] and Watson for Oncology [25]. The knowledge base systems provide decision support through a knowledge base, inference engine, and human–machine interface. The non-knowledge base systems adopt artificial intelligence methods to summarize and clarify knowledge during human–machine interaction and continuous training and provide suggestions to users. Knowledge base systems have higher accuracy but cannot fill in data, while the opposite is true for non-knowledge base systems. However, both methods provide decision support for users through third-party knowledge without considering the value orientations or preferences of users.

**Fig. 1:**
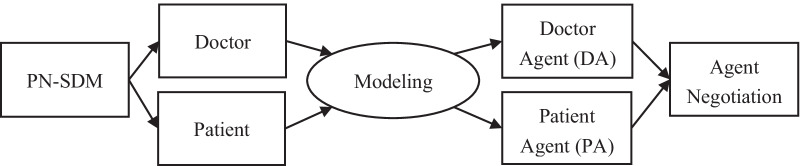
Problem conversion

Therefore, focusing on the above two limitations, this paper concerns the behavior and preferences of the doctor and patient and proposes an intelligent preferences negotiation and assistant decision-making method. For SDM, the committed steps are as follows: (1) doctor inform patient for whom a decision needs to be made and value the opinions of patient; (2) doctor explain the options and their merits and demerits; (3) doctor and patient discuss the preferences of patient and support patient in thinking carefully; and (4) doctor and patient discuss the decision wishes of patient, make or postpone decisions, and discuss follow-up [26]. According to the third point in this definition, doctor and patient are the most obvious contacts in SDM, and they need to integrate preferences related to issues of mutual concern. Therefore, the problems of preference negotiation in SDM (PN-SDM) can be seen as interactive problems formed by doctor and patient. As independent individuals in this interactive process, doctor and patient can share information partially but not fully. Each side has its own knowledge, beliefs, obligations, and intentions, can communicate with others, and is managed independently but is affected by the other parties and constrained by society. The above factors can be matched with the definition of an agent in “Intelligent Agents: Theory and Practice” [27], written by Wooldridge in 1995. Hence, as shown in Fig. 1, we can regard the direct participants, doctor, and patient in the PN-SDM as agents that are independent, interrelated, and restrained by the environment. Moreover, we can regard the communication of preferences between doctor and patient as the negotiation between agents. Accordingly, we can solve the problems of the PN-SDM by solving the preference negotiation problems between agents.

Agent-based auto-negotiation technology is a basic and useful method for cooperative problem-solving. It has been widely developed and used in the fields of resource allocation and task solving [28, 29], e-commerce [30], cloud computing [31, 32], etc. From a technical point of view, the research on agent negotiation can be summarized into four types: game theory-based agent negotiation [33, 34], heuristic-based agent negotiation [35, 36], argumentation-based agent negotiation [37], and distributed constraint-based agent negotiation [38, 39]. Of the four approaches, the game theory-based agent negotiation method provides some descriptive concepts for the optimal solution, but the calculation method is unclear. Although heuristic-based agent negotiation relaxes the completely rational setting compared with the game theory-based agent negotiation method, the negotiation result is not optimal. It is impossible to cover the whole negotiation space with this method. Argumentation-based agent negotiation can overcome various problems caused by the lack of information exchange in the negotiation process. However, the design of the negotiation protocol, communication language, and information identification are more complex. Distributed constraint-based agent negotiation is an effective method for agents to reach an agreement and can ensure Pareto-optimal negotiation and the lowest information disclosure.

Considering the advantages and disadvantages of the above methods, we aim to develop a negotiation model based on the theory of distributed constraint to solve the problems of PN-SDM. In addition, it provides a treatment recommendation framework to assist doctors and patients in SDM based on their preferences. From a theory perfection perspective, it is the first attempt to provide an agent-based PN-SDM method of realizing PN-SDM intelligence and quantifying preference information. In terms of actual application, it helps to improve the efficiency of negotiation in SDM, provide treatment plans reference of decision-making, and promote the clinical application of SDM.

## Methods

### Modeling the PN-SDM as a DFCSP

**Fig. 2:**
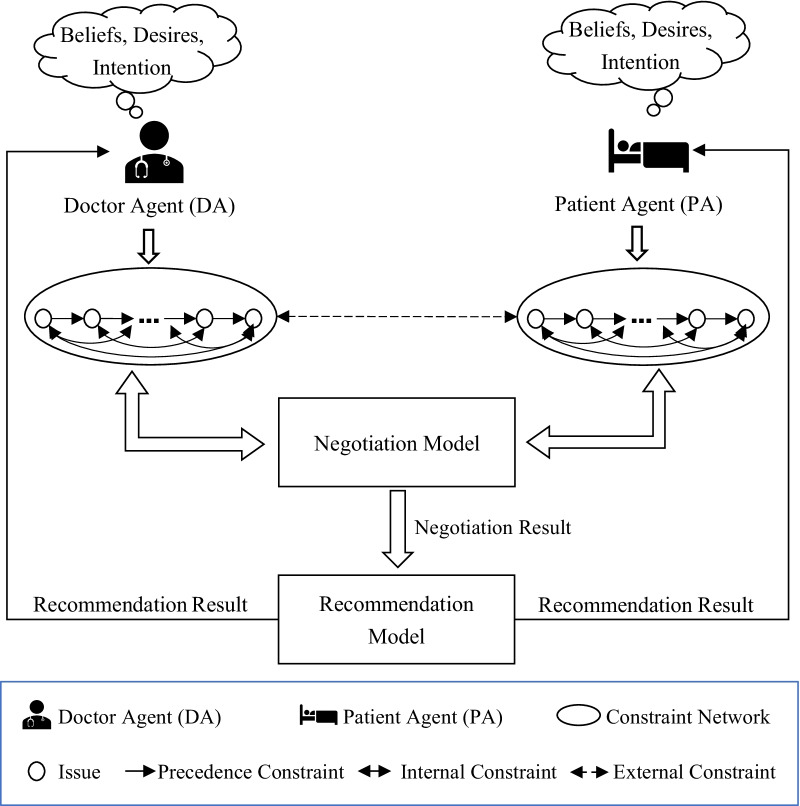
Agent-based negotiation for the bilateral PN-SDM

For SDM, PN-SDM problems involve doctor and patient discussing preferences for multiple issues related to treatment. In the process of bilateral PN-SDM, doctor and patient express their own value orientation and negotiation in the form of iterative interaction. Based on the description in the background section, the PN-SDM is modeled as an agent-based PN-SDM, and it can solve PN-SDM problems by resolving conflicts between the doctor agent (DA) and patient agent (PA). Accordingly, the exchange of an offer and counteroffer is the interaction form between agents in agent-based PN-SDM. As shown in Fig. 2, negotiation for treatment issues in PN-SDM can be modeled as a triple $({\mathcal {D}}, {\mathcal {P}}, {\mathcal {I}})$, where ${\mathcal {D}}$ and ${\mathcal {P}}$ represent DA and PA, respectively, and ${\mathcal {I}}$ is the interrelationship between the two types of agents. In addition, we structure a recommendation framework that transforms the negotiation results into treatment plans to assist SDM.

In the practical PN-SDM, a great deal of uncertain and imprecise information exists. For example, for doctor and patient, the satisfaction and priorities of different issues are imprecise, the aggregated satisfaction for all issues is uncertain, and the influence of the opponent in the negotiation process is unclear. That is, in the auto-agent negotiation of the PN-SDM, there are many possible constraints between agents and between issues that cannot be accurately obtained by each other but can directly affect the final negotiation result. Therefore, we need to improve the negotiation efficiency of the DA and PA by using imprecise and uncertain information effectively to acquire knowledge. Some studies have shown that fuzzy theory aids proper handling of uncertainty [40]. From this perspective, we can model these incomplete, uncertain, and interlinked information as fuzzy constraints to acquire and express knowledge as flexibly as possible. Then, the PN-SDM problems can be represented as a distributed fuzzy constraint satisfaction problem (DFCSP) that causes a mutually acceptable negotiation result received by both the DA and the PA. In addition, the behavior model of agents can be built by fuzzy constraints. For the PN-SDM, the constrained interrelation between agents determines whether there is a solution that satisfies all the constraints of DFCSPs. A DFCSP can be expressed as a distributed fuzzy constraint network in which fuzzy relationships are assigned by each agent and among agents. A distributed fuzzy constraint network can be defined as follows:

#### Definition 1

A distributed fuzzy constraint network (*U*, *X*, *C*) in an PN-SDM $({\mathcal {D}}, {\mathcal {P}}, {\mathcal {I}})$ can be defined as a set of fuzzy constraint networks $\{N^1,\ldots ,\ N^l,\ldots ,\ N^L\}$, where $N^l=(U^l, X^l, C^l)$ is from agent *l*. There are two kinds of fuzzy constraint networks in the PN-SDM, that is, $N^{{\mathcal {D}}}=(U^{{\mathcal {D}}}, X^{{\mathcal {D}}}, C^{{\mathcal {D}}})$ and $N^{{\mathcal {P}}}=(U^{{\mathcal {P}}}, X^{{\mathcal {P}}}, C^{{\mathcal {P}}})$, which belong to the DA and PA, respectively. Where

$U^l$ is the universe of discourse for the fuzzy constraint network, $N^l$;

$X^l=(U_{i=1}^nX_i^l)$ is a tuple of all non-recurring objects of an agent in a fuzzy constraint network; and

$C^l=(U_{i=1}^nX_i^l)$ is a set of all fuzzy constraints in the fuzzy constraint network, which includes the internal constraints among objects in *X* and external constraints between an agent and its opponent.

*U* is the universe of discourse for a distributed fuzzy constraint network;

$X=(U_{l=1}^LX_l)$ is a tuple of all non-recurring objects in the distributed fuzzy constraint network; and

$C=(U_{l=1}^LC_l)$ is a set of all fuzzy constraints in the distributed fuzzy constraint network.

As stated in Definition 1, non-recurring objects $X^l$ represent the beliefs, environmental cognition (e.g., deadline and medical resources), and other attributes of agent *l*. Fuzzy constraint set $C^l$ includes all constraints of agent *l*, for instance, priority constraints (e.g., the priority of issues), objective constraints (e.g., the desire of cost, effectiveness, and other objectives), and constraints with other agents. The solution to $X^l$, a fuzzy constraint network, can be seen as intention $\Pi _{N^l}$, which expresses that fuzzy set $X^l$ of non-recurring objects satisfies all fuzzy constraints $C^l$. The $\Pi _{N^l}$ of agent *l* can also be regarded as $\Pi _l$. Given issues set $I=\{I_1,I_2,\ldots ,I_i,\ldots ,I_n\}$ and feasible solution $S\epsilon \Pi _l$,the aggregated satisfaction value (ASV) for *S* of agent *l* is:

1 $$\begin{aligned} \Psi \left( S\right) =\sum _{i=1}^{n}{w_i*F_i(S)} \end{aligned}$$

where $F_i\left( S\right)$ is the $i_{th}$ fuzzy membership function of *S*. It can be obtained directly through the doctor and patient and will flexibly and effectively represent their preferences on certain issues. *n* is the number of issues that need to be negotiated by the DA and PA, and $w_i$ is the corresponding weight factor for issue $i_{th}$.

### Negotiation and recommendation model for PN-SDM

The negotiation and recommendation models for the PN-SDM are presented in this section. The negotiation model of the PN-SDM is named the fuzzy constraint-directed agent-based negotiation (FCAN) model, which includes the behavior framework of the DA and PA and the negotiation protocol by which negotiators need to abide. The recommendation model of the PN-SDM is mainly used to transmute the negotiation result into specific treatment plans for doctor and patient to choose and reference. Furthermore, we introduce an instance to illustrate the mechanism of negotiation and recommendation models of agent-based PN-SDM in detail.

#### Negotiation model for PN-SDM

The presented FCAN model for PN-SDM is mainly composed of the negotiation process and the negotiation protocol.

*Negotiation process* Within the limits of time, the DA and PA will comply with the negotiation protocol to solve their own DFCSP through the exchange of an offer and counteroffer. The FCAN model provides a negotiation framework for the DA and PA. The agent negotiation steps include concession calculation, feasible set generation, offer generation, and negotiation terminals. Before negotiation terminates, the above process will be repeated.

*Step 1 (Concession calculation)* For an agent, the response state of its opponent, its internal state, and the environmental state reflect the intention of the opponent, the desire of the agent, and the environmental constraint of the agent. Therefore, the agent can decide whether to concede and determine the value of the concession by evaluating the three states.

The opponent’s responsive state ${\textbf {O}}$ represents the degree of difference between the last offer *A* and the most recently received counteroffer *B*, which can be defined as follows:

2 $$\begin{aligned} \sigma =1-(G\left( A_0,B_0\right) -G\left( A,B\right) )/G\left( A_0,B_0\right) \end{aligned}$$

where $A_0$ is the initial offer and $B_0$ is the first counteroffer from its opposing agent. $G\left( A, B\right)$ is the distance measurement of offer *A* and counteroffer *B* on issue $I_i\epsilon X$. The calculation formula is as follows:

3 $$\begin{aligned} G\left( A,B\right) =\frac{\sqrt{\sum _{i=1}^{n}{L(A_i,B_i)}^2}}{n} \end{aligned}$$

where $A_i$ and $B_i$ are the possibility distributions of offer *A* and counteroffer *B* for issue $I_i\epsilon I$, respectively.

The internal state ${\textbf {I}}$ of the agent includes the satisfaction level $\rho$ and the tightness $\delta$ related to the last offer *A* and a set of alternative solutions:

4 $$\begin{aligned} \rho= & {} \Psi (S^*) \end{aligned}$$

5 $$\begin{aligned} \delta= & {} 1-(\rho -\varepsilon ) \end{aligned}$$

where, $S^*\in \Pi$ is the prospective solution of the agent in the last negotiation round, $\Psi \left( S^*\right)$ is the agent’s satisfaction with $S^*$, and $\varepsilon$ is the aggregated satisfaction threshold.

In the PN-SDM, the major environmental constraints ${\textbf {E}}$ of the DA and PA are time constraints. Thus, we can use a function [41] to describe the constraints of time:

6 $$\begin{aligned} t={k+\left( 1-k\right) \left( \frac{r_{now}}{r_{max}}\right) }^\beta \end{aligned}$$

where $r_{now}$ is the current negotiation round, $r_{max}$ is the deadline of negotiation, and parameter *t* represents the constraint of negotiation time. *k* and $\beta$ in formula (6) are constant, while $1>\beta >0$ and $0\le k\le 1$.

Through Eqs. (2–6), we can obtain the responsive state ${\textbf {O}}=\{\sigma \}$ of the opponent, internal state ${\textbf {I}}=\{\rho ,\ \delta \}$ of the agent, and environmental state ${\textbf {E}}=\{t\}$. Then, the concession value $\Delta \varepsilon$ of the agent when it negotiates can be calculated as follows:

7 $$\begin{aligned} \Delta \varepsilon =\left( \mu _\rho \left( \rho \right) \ \Lambda \ \mu _\delta \left( \delta \right) \ \Lambda \ \mu _\sigma \left( \sigma \right) \ \Lambda \ \mu _t\left( t\right) \ \right) ^\omega \end{aligned}$$

where $\mu _\sigma (\sigma )$, $\mu _\rho (\rho )$, $\mu _\delta (\delta )$ and $\mu _t(t)$ denote the desire for concession about the degree of difference, satisfaction level, degree of tightness, and time constraint, respectively. Parameter $\omega$ is associated with the negotiation strategy, and it can adjust the negotiation strategy used in the calculation of the concession value, namely, (i) collaborative strategy, when $\omega <1$; (ii) win-win strategy, when $\omega =1$; and (iii) competitive strategy, when $\omega >1$.

Given the concession value $\Delta \varepsilon$ and the behavior state $\varepsilon$ in the last round, the new behavior state $\varepsilon ^*$ of the agent can be calculated by the following:

8 $$\begin{aligned} \varepsilon ^*=\varepsilon -\Delta \varepsilon \end{aligned}$$

*Step 2 (Feasible solution generation)* Given a fuzzy constraint network *N*, intension $\Pi$, and new behavior state $\varepsilon ^*$, feasible solutions can be acquired. The definition of a feasible solution *P* is as follows:

9 $$\begin{aligned} P=\Gamma (\Pi , \varepsilon ^*)=\{S|(S\epsilon \Pi )\Lambda (\varepsilon \ge \Psi \left( S\right) \ge \varepsilon ^*)\} \end{aligned}$$

where $\Psi \left( S\right)$ is the ASV of the agent about objectives. Given counteroffer *B* and feasible solution *P*, the selection of prospective solution $S^*$ is as follows:

10 $$\begin{aligned} S^*=arg({max}_{S\in P}H(S,B)) \end{aligned}$$

where *H*(*S*, *B*) is a utility function that is used to evaluate the preference for feasible solution $S\epsilon P$ and the similarity between counteroffer *B* and feasible solution *S*. Its definition is as follows:

11 $$\begin{aligned} H\left( S,B\right) =\frac{1}{n}\sqrt{\sum _{i=1}^{n}{(W_1{(S_i)}^{\omega _1}\wedge W_2{(S_i,B_i)}^{\omega _2})}^2} \end{aligned}$$

where $W_1$ is a preference function on issue *i*, and $W_2$ is a similarity function that measures the preference distance between solution *S* and counteroffer *B* on the $i_{th}$ issue. Parameters $\omega _1$ and $\omega _2$ are weights related to preference and similarity, respectively. The different negotiation strategies represent different negotiation behaviors related to the selection of solutions and the speed of conflict resolution. Thus, the parameters associated with various negotiation strategies are defined as follows:

Collaborative strategy: $$\omega _1\le 1.0$$, $$\ \omega _2\le 1.0$$, $$\ \omega _1<\omega _2$$

Win–win strategy: $\omega _1\le 1.0$, $\ \omega _2\le 1.0$, $\ \omega _1=\omega _2$

Competitive strategy: $$\omega _1\ge 1.0$$, $$\ \omega _2\ge 1.0$$, $$\ \omega _1>\omega _2$$

With the collaborative strategy, an agent tends to reach an agreement in the least time, and it will concede to a solution that captures more benefits for the opposing agent. In a negotiation using the win-win strategy, the negotiation result will satisfy the desire of both parties if the agent fully cares about its own interests and those of its opponent. The agent will care only about its own interests during the negotiation if the competitive strategy is adopted.

*Step 3 (Offer generation)* Given feasible solution *P* and prospective solution $S^*$, the generation of a new offer $A^*=\{A_1^*,A_2^*,\ldots ,A_i^*,\ldots ,A_n^*\}$ for a set of issues $I\epsilon X$ is defined as:

12 $$\begin{aligned} A^*=arg({max}_{S\in S^*}\ S(S,\ B)) \end{aligned}$$

where *S*(*S*, *B*) is a similarity function that is used to evaluate the similarity between counteroffer *B* and prospective solution *S* on the value of issues, and its formula is:

13 $$\begin{aligned} S(S,\ B)=\frac{1}{n}\sum _{i=1}^{n}{exp\{-k|S_i-B_i|\}} \end{aligned}$$

In formula (13), *k* is a constant, and $k>0$, $S_i$ and $B_i$ are the values of *S* and *B* on issue *i*.

*Step 4 (Negotiation terminal)* The negotiation will be continued until the agent reaches an agreement or until no new offer or counteroffer is generated. Given feasible solution *P* and counteroffer *B*, negotiation will be terminated with two states, one of which is agreed upon and the other of which fails, where the condition of a successful negotiation is as follows:

14 $$\begin{aligned} \Psi \left( B\right) \ge \varepsilon ^*\ \end{aligned}$$

and the condition of a failed negotiation is as follows:

15 $$\begin{aligned} \varepsilon ^*\le 0\ or\ P=\emptyset \end{aligned}$$

If the ASV of the agent to counteroffer *B* is greater than the aggregated satisfaction threshold $\varepsilon ^*$ in the new round, this indicates that the agent agrees to accept the offer from its opponent and that DFCSPs are solved because the DA and PA reach an agreement. Otherwise, the negotiation is terminated in a failed state, possibly because the new satisfaction threshold is less than zero or the set of feasible solutions is empty.

*Negotiation protocol* The negotiation protocol is the definition, representation, processing, and semantic explanation of agent communication language, which is mainly used to deal with interactions between agents during negotiation. In fact, it is the rule by which all agents must abide. It defines all interactions among agents and determines the sequence and structure of messages. Concerning the problems of PN-SDM, the DA and PA can negotiate by sending and receiving various types of messages, including the following:

*Ask* (negotiator, opponent, offer) the DA sends an offer to its opponent to query the value of the issues concerning the treatment plan.

*Tell* (negotiator, opponent, counteroffer) the PA transmits the counteroffer to its opponent.

*Accept* (negotiator, opponent, offer) the negotiator accepts the offer presented by its opponent and terminates the negotiation.

*Reject* (negotiator, opponent) the negotiator sends a rejection message to its opponent if there are no offers sent to the opponent.

*Agree* (negotiator, opponent, counteroffer) the negotiator temporarily accepts the offer from its opponent and waits for the affirmation of the opponent.

*Abort* (negotiator, opponent) the negotiator aborts this negotiation for certain reasons.

**Fig. 3:**
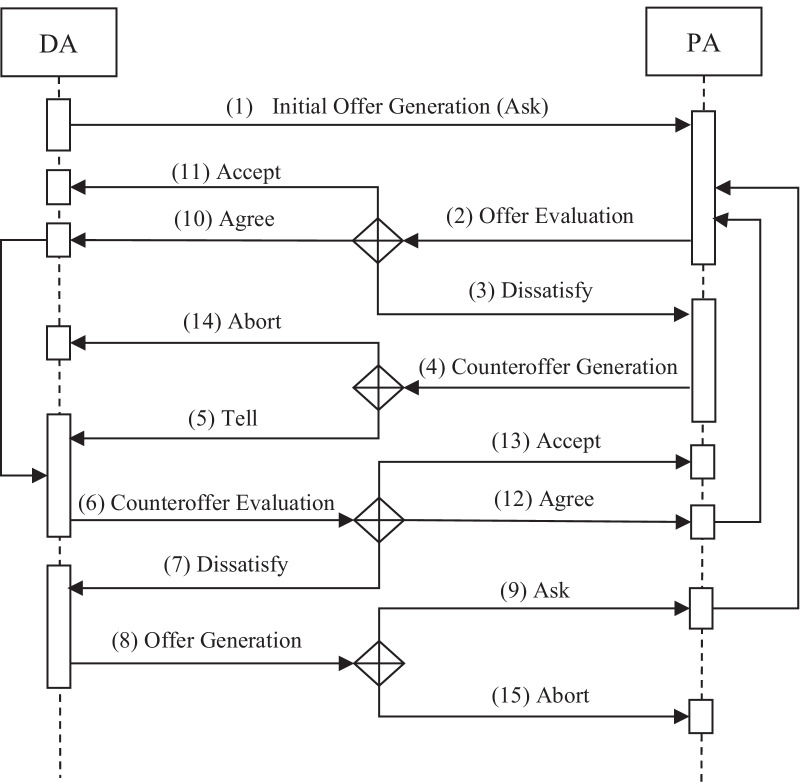
Negotiation process between the DA and the PA

In the formal negotiation process, as shown in Fig. 3, the DA will first send the *Ask* () message with an initial offer to the PA. When the offer is received from the DA, the PA evaluates the offer to determine whether this initial offer meets its own constraints. If the constraints are not met, then a counteroffer is generated according to formulas (2–13), and a *Tell* () message is sent to the DA. When the DA receives the PA’s counteroffer, it needs to evaluate and check whether it meets the negotiation requirements of the DA. If the counteroffer does not meet these requirements, then the concession value will be calculated, and an offer will be generated by the DA. Then, the DA will readjust the requirements for the negotiation items in step 7 and send an *Ask* () message with a new offer to the PA in step 8. Next, an exchange of the offer and counteroffer between the DA and PA will be carried out iteratively according to steps 2–9.

When the offer or counteroffer for all negotiated issues is accepted by the agent in step 2 or step 6, an *Agree* () message will be sent to the opponent’s agent. Then, in steps 11 or 13, if and only if the solution set of all negotiation issues satisfies formula (14), that is, all received messages are *Agree* (), the agent will send an *Accept* () message to its opponent, which means that the negotiation has reached an agreement. Otherwise, the DA or PA will send an *Abort* () message to its opponent, and the negotiation will fail because the satisfaction threshold of both parties is too low or the feasible solution set of an agent on some negotiation issues is null. Of course, there is another situation that also represents negotiation failure: the current negotiation time exceeds the agreed-upon negotiation time. In the negotiation process between the DA and the PA, if the negotiation time does not exceed the specified deadline, then both parties will continue to negotiate. However, if it exceeds the deadline, then the negotiation will be terminated in a failed state.

#### Recommendation model for PN-SDM

The negotiation result between a DA and a PA is an agreement on the value of all issues’ preferences, such as the approximate value of treatment cost, the estimated value of effectiveness, and the possible value of side-effects. However, the motivation of PN-SDM is to better complete SDM in clinical practice. Moreover, it aims to service for obtaining treatment plans that meet the preferences of both sides and conform to the actual patient’s condition. Therefore, we propose a recommendation model that can transform the negotiation result into feasible treatment plans and accomplish the recommendation of treatment plans.

**Fig. 4:**
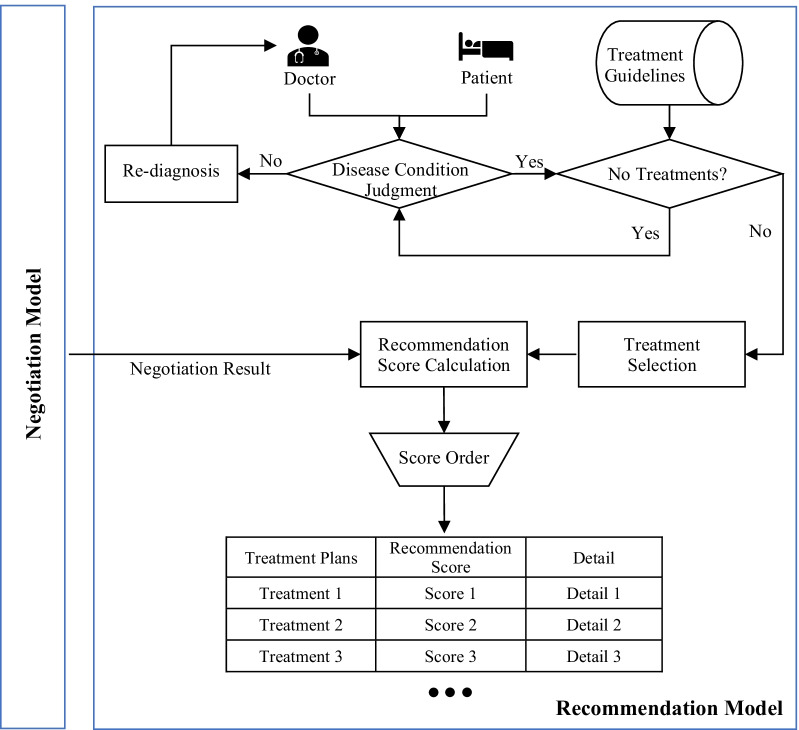
Treatment recommendation model for the PN-SDM

In this model, first, basic information is shared between a doctor and a patient, and the patient’s condition is diagnosed by the doctor. Then, treatment plans that fit the patient’s condition are selected from the treatment guidelines. Next, the recommendation scores between selected treatment plans and the negotiation result are calculated, and the scores are then sorted. Finally, treatment plans with recommendation information are sent to agents. Of course, the recommendation results are reasonable and explainable. The concepts related to this recommendation model are shown in Fig. 4.

Suppose that a DA and a PA agree on the value of each issue through negotiation. Then, it is necessary to calculate the similarity value between solution $S^*$ and each treatment plan and match the solution with each treatment plan chosen. The recommendation is defined as follows:

16 $$\begin{aligned} \Phi \left( S\right) =\sum _{i=1}^{N_i}{w_i*S_i\left( {\widetilde{S}}_i\right) } \end{aligned}$$

where *n* is the number of negotiation issues, i.e., negotiation objects, and $w_i$ is the weight of the relevant issues concerning treatment plans (e.g., a DA’s issue preference weight, a PA’s issue preference weight, or the average issue preference weight of the DA and PA). In formula (16), ${\widetilde{S}}_i$ is the possibility distribution of the value of $S^*$ on the $i_{th}$ issue, and $S_i\left( {\widetilde{S}}_i\right) \in [0,1]$ is the similarity calculation of the negotiation issue level, that is, the fuzzy membership function corresponding to each negotiation issue related to treatments. Based on this, we can obtain the final list of recommended treatment plans.

#### An illustration

In clinical practice, the PN-SDM applies to the treatment decision-making method for various chronic diseases. For example, in the PN-SDM for childhood asthma, the main task of doctor and patient is to negotiate with each other and integrate the preferences of cost, effectiveness, side-effects, risk, and convenience, which are issues of concern for both negotiation parties. Therefore, based on agent technology, taking the PN-SDM concerning childhood asthma as an example, our bilateral multi-issue negotiation model is adopted to negotiate the issues involved in the treatment plan to solve the PN-SDM problems.

This section provides a case to illustrate how the proposed negotiation and recommendation models work for the asthma PN-SDM. We present an example of an PN-SDM, a case of an 8-year-old child whose asthma severity reached grade 4. By obtaining the preferences of doctor and patient for each issue and applying the FCAN model, the DA and PA can be constructed to solve the PN-SDM problems and better complete the PN-SDM. Notably, the preferences of doctor and patient are obtained by the questionnaire. In this case, the negotiated issues include cost (0–8 thousand RMB), effectiveness (1–10 rank), side-effects (0–100 %), risks (0–100 %), and convenience (1–10 rank). The value range of each issue is given under the guidance of medical experts to restrict the solution space.

In the negotiation process, the preferences of the DA and PA will be different due to the different considerations of doctor and patient. These preferences can be constructed into fuzzy membership functions as described above. In this case, all preferences (e.g., the preference of value and weight) of DA and PA regarding every issue can be set as in Tables 1 and 2, respectively. Indeed, these preferences may change with information sharing between DA and PA. However, changeable preferences will affect the quality and speed of negotiation and decision. Thus, we assume negotiations are conducted on the premise of parties’ preferences fixed. If the preferences change, the negotiation can be restarted.

**Table 1:** All preferences of DA

Issues preferences	Value range preference	Minimum preference value	Maximum preference value	Weight preference
Cost	4–8	3.5	8	0.15
Effectiveness	7–8	5	10	0.3
Side-effects	0.1–0.15	0.01	0.25	0.25
Risk	0.1–0.15	0.05	0.25	0.2
Convenience	7–8	6	10	0.1

**Table 2:** All preferences of PA

Issues preferences	Value range preference	Minimum preference value	Maximum preference value	Weight preference
Cost	1–3.5	0	4.5	0.3
Effectiveness	9–10	8	10	0.25
Side-effects	0.0–0.05	0.0	0.1	0.2
Risk	0.0–0.05	0.0	0.1	0.15
Convenience	9–10	8	10	0.1

For this negotiation, the initial satisfaction thresholds of the DA and PA are set to 1.0, the reservation values of satisfaction are all 0.0, and the negotiated issues concerning treatment are cost, effectiveness, side-effects, risk, and convenience. To ensure successful PN-SDM within a small number of rounds, suppose that the DA and PA tend to reach an agreement as quickly as possible. That is, the collaborative negotiation strategy (i.e.,$\ \omega _1<\omega _2$; $\omega _1$, $\ \omega _2\le 1.0$) is used for the DA and PA in the illustrative case. According to Eqs. (14) and (15), the negotiation is terminated when the DA reaches an agreement with the PA or a negotiator withdraws from the negotiation. To summarize this negotiation, the data changes for the DA and PA are given in Tables 3 and 4.

**Table 3:** Changes in the DA’s data during the negotiation

Round	Concession value	Threshold	Feasible set size	Prospective set size	Offer	ASV
1	0	1	2	1	[4.0, 7, 0.1, 0.1, 7]	1
2	0.0285	0.9593	48	16	[3.98, 8, 0.1, 0.1, 8]	0.9940
3	0.0480	0.8942	256	4	[3.95, 8, 0.09, 0.09, 8]	0.9172
4	0.0710	0.8017	288	8	[3.9, 8, 0.08, 0.09, 8]	0.8744
5	0.1000	0.6758	252	4	[3.84, 8, 0.08, 0.08, 8]	0.8164
6	0.1272	0.5195	720	8	[3.83, 8, 0.18, 0.08, 8]	0.7940
7	0.1585	0.3289	3300	2	[3.79, 9, 0.07, 0.07, 9]	0.5337

**Table 4:** Changes in the PA’s data during the negotiation

Round	Concession value	Threshold	Feasible set size	Prospective set size	Offer	ASV
1	0	1	2	1	[1.0, 9, 0.0, 0.0, 9]	1
2	0.0287	0.9591	10	2	[3.54, 9, 0.05, 0.05, 9]	0.988
3	0.0446	0.8982	14	1	[3.6, 9, 0.05, 0.05, 9]	0.97
4	0.0598	0.8189	18	1	[3.68 9, 0.06, 0.06, 9]	0.876
5	0.0835	0.7119	23	2	[3.69, 9, 0.06, 0.06, 9]	0.873
6	0.0961	0.5904	104	2	[3.79, 9, 0.07, 0.07, 9]	0.7730

In these tables, the concession value $\Delta \varepsilon$, threshold $\varepsilon ^*$, the size of feasible set *P*, and the size of prospective set $S^*$ are listed. In addition, the offer for each round of DA and PA, as well as the relevant ASV, are shown in the tables. Each activity is specified by the expected value of the issues. Through the detailed negotiation steps described previously, DA and PA finally reach an agreement in the 7th round of negotiation, that is, [Cost: 3.79, Effective: 9, Side-effects: 0.07, Risk: 0.07, Convenience: 9]. In the last round, the ASV of DA is greater than its threshold, which satisfies Eq. (14). Finally, the ASVs of DA and PA with the offer are 0.5337 and 0.7730, respectively.

According to the age of asthmatic children and the severity of asthma, feasible treatments for children can be obtained by the guidelines of bronchial asthma in children [42], as shown in Table 5. Table 5 presents the corresponding relationships between the four optional treatment options and the value of the issues under the set scenario. In clinical practice, this table is given based on big data analytics and medical experts’ opinions. In fact, it is not universal and only used for the experimental data of the case in reference. However, based on this, we can match the negotiation results of the DA and PA with the selected treatment plans determined by the children’s age and asthma control level. Table 6 shows the three possible pieces of information on the weight of each issue in formula (16), which is used for the similarity calculation with treatment plans.

**Table 5:** Range of treatment on issues

Treatments issues	Cost	Effectiveness	Side-effects	Risk	Convenience
En-high dose ICS/LABA^a^	2.7–4.5	8–9	1–1.5	1–2	9.5–10
En-high dose ICS^b^ + LTRA^c^	4.3–6.5	7–8	2-3	1.5–2.5	9–9.5
En-high dose ICS + sustained-release THP^d^	2–4.2	6-7	6-10	2–2.5	8–8.5
En-high dose ICS/LABA + LTRA	5.7-7.3	9-10	5-6	1-1	7.5–8
En-high dose ICS/LABA + sustained-rTHP	3.5–5	9–10	6–8	1–1	7.5–8

^a^A combination of inhaled corticosteroids and long-acting beta2-agonists

^b^Inhaled corticosteroid

^c^Leukotriene receptor antagonists

^d^Theophylline

**Table 6:** Three kinds of weights related to treatment

Weights issues	Cost	Effectiveness	Side-effects	Risk	Convenience
Issues weight of the DA	0.15	0.3	0.25	0.2	0.1
Issues weight of the PA	0.3	0.25	0.2	0.15	0.1
Avg. weight of the DA and PA	0.225	0.275	0.225	0.175	0.1

**Fig. 5:**
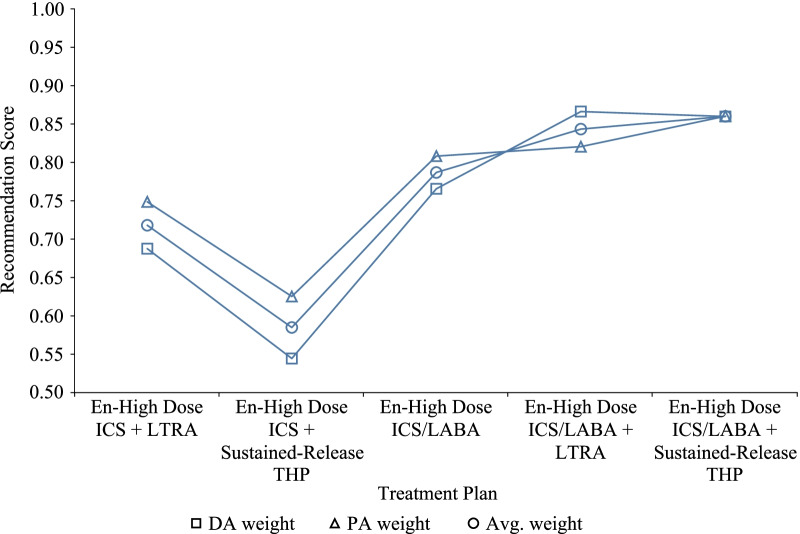
Similarity between negotiation results and treatments of the FCAN with collaborative strategy

Referencing the content shown in Tables 5 and 6 and according to our recommendation model, the similarity value, that is, the recommendation score of the negotiation result and each treatment plan, can be calculated, as shown in Fig. 5.

In the case of fully weighing the three possible weights, the similarity value between the negotiation result and the treatment plans is different under scenarios with different weight information. However, overall, the order of similarity (e.g., recommendation score) between the negotiation result and the treatment plan is consistent, as follows:

En-high dose ICS / LABA + Sustained-release THP $\succ$ En-high dose ICS / LABA + LTRA $\succ$ En-high dose ICS / LABA $\succ$ En-high dose ICS + LTRA $\succ$ En-high dose ICS + Sustained-release THP

This result has been fully affirmed by doctor and patient. From the above description, the treatment plans recommended by our recommendation model are consistent regardless of whether they are based on DA preferences, PA preferences, or a mix of both. It indicates that the recommendation results obtained by our recommendation model are unambiguous from any point of view.

## Results and discussion

### Experimental setup

*Objective* To better verify the efficiency of our negotiation model, performance comparisons of different strategies and different negotiation pairs (that is, different doctor and patient) are demonstrated in this section. The evaluation indicators include the combined ASV, running time in seconds, and negotiation rounds. Be noted that there are few agent-based methods to solve the PN-SDM problems. Thus, the comparative experiments of this paper are limited.

*Environment* The program was written and compiled by Java. In addition, the behavior framework of agents and the negotiation between agents in this article are all provided by the Genius [43] platform.

*Dataset* The problems of PN-SDM are common medical preferences problems that involve a number of issues and negotiator pairs. Thus, taking childhood asthma as an example, the negotiation issues include cost, effectiveness, side-effects, risk, convenience, treatment time, prognosis, recurrence rate, and complications. The preference data of doctor and patient on issues were collected by questionnaires in the Department of Pediatrics at Xiamen Hospital of Traditional Chinese Medicine. The preferences data consist of the value preferences and weight preferences of the issues.

*Parameter setup* In the experiments, the numbers of DAs and PAs in a negotiation are all set to 1. The maximum negotiation round is set to 15 (if the negotiation rounds of the DA and PA, that is, doctor and patient, exceed 15, then the negotiation fails). We set the preference weight of each issue of the DA and PA to the same value to effectively verify the problem-solving ability of our negotiation model when the number of issues increases. In addition, all the experimental results in this paper are the average values after 200 repeated experiments.

### Performance comparisons among different FCAN strategies

Different negotiation strategies represent different negotiation behaviors. To evaluate the impact of various negotiation strategies for PN-SDM problems, including collaborative, win-win, and competitive, different issue numbers are used to compare performance. The negotiation strategies of the FCAN model are related to parameters $\omega _1$ and $\omega _2$ in formula (11), so in the experimental process, the strategy parameters are set to (i) a collaborative strategy, when $\omega _1=0.8$ and $\omega _2=1$; (ii) a win-win strategy, when $\omega _1=\omega _2=1$; and (iii) a competitive strategy, when $\omega _1=1.2$ and $\omega _2=1$.

To test the performance of our negotiation model with different strategies when the problem size increases, the performance of the three negotiation strategies is compared with the Time model [41] when the number of issues increases. In the experiment, the number of negotiation issues is changed (the initial number of issues is set to 1, and two issues are added every time) to simulate different PN-SDM scenarios.

**Table 7:** Performance comparisons in terms of combined ASV

Number of issues	Time	FCAN		
		Collaborative	Win–win	Competitive
1	1.33	1.34	1.35	**1.36**
3	1.3522	1.3622	1.3622	**1.3785**
5	1.2906	1.3053	1.3113	**1.3113**
7	1.1512	1.1624	1.2133	**1.2167**
9	1.1462	1.1575	1.1583	**1.1722**

The data marked in bold indicates the best performance

The changes in the combined ASV values of the DA and PA using different strategies during the process of increasing the number of negotiation issues from 1 to 9 are represented in Table 7. It can be seen from this table that with the increase in negotiation issues, the trend of combined ASV values of the DA and PA is not fixed. This means that with the increase in the number of negotiation issues, the combined ASV obtained by the DA and PA may increase or decrease. On each negotiation issue scale, we know that the combined ASV value of the Time model is less than that of the FCAN model with any negotiation strategy. For FCAN, it achieves better performance with a competitive strategy than a win-win strategy or a collaborative strategy in terms of the combined ASV and when the number of issues increases. The data of FCAN with a competitive strategy are marked in bold in this table. However, the FCAN with a competitive strategy requires more running time and negotiation rounds, as shown in Tables 8 and 9.

**Table 8:** Performance comparisons in terms of running time

Number of issues	Time	FCAN		
		Collaborative	Win–win	Competitive
1	**0.0243**	0.0369	0.0364	0.0373
3	**0.0252**	0.0386	0.0390	0.0396
5	**0.0279**	0.0549	0.0445	0.0447
7	**0.0289**	0.1942	0.1552	0.1800
9	**0.0291**	3.8711	3.2503	1.7457

The data marked in bold indicates the best performance

**Table 9:** Performance comparisons in terms of negotiation rounds

Number of issues	Time	FCAN		
		Collaborative	Win–win	Competitive
1	9	**5**	6	7
3	10	**6**	7	9
5	10	**7**	8	9
7	11	**7**	8	10
9	11	**8**	9	10

The data marked in bold indicates the best performance

The running time and negotiation rounds required by the DA and PA with different negotiation strategies of the FCAN model when the number of negotiation issues increases from 1 to 9 are compared in Tables 8 and 9. The data marked in bold in these tables indicates the best performance. With the increase in the number of issues, the number of running time and negotiation rounds all gradually increase, which is more in line with reality. When the scale of the issue increases, both sides need to spend more time negotiating to reach an agreement. However, no matter how the running time increases, it can be counted in seconds, and regardless of how much the number of negotiation rounds increases, it will not exceed the maximum number of negotiation rounds. At the same time, the number of negotiation rounds required for different negotiation strategies can be ranked as Time > FCAN with competitive > FCAN with Wwin–win > FCAN with collaborative, which is in line with actual negotiation. Although the negotiation running time of Time is less than that of the FCAN model, our negotiation run time is no more than one minute. During the negotiation process, our presented model will search the possible solution space, resolve conflict between the DA and the PA, and finally find the optimal solution for solving PN-SDM problems. However, human beings will still be free from complex and trivial preference negotiation tasks with our models.

Tables 7, 8 and 9 reveal that the performance of the FCAN model is better than that of the Time model in terms of combined ASV and negotiation rounds. Although the FCAN model needs more time to explore the solution space, the running time of FCAN is still a few seconds. For FCAN, the competitive strategy results in the highest combined ASV but the highest number of negotiation rounds. The collaborative strategy results in the lowest combined ASV and number of negotiation rounds. The value of the combined ASV obtained and negotiation rounds required for the win-win strategy are somewhere in between. In addition, the running time of FCAN with a win-win strategy is often less than that of FCAN with a collaborative strategy or competitive strategy. The purpose of negotiation is to obtain the highest possible result for combined ASV with the lowest possible running time and fewest negotiation rounds. Therefore, the overall performance of the win-win strategy is best when the number of negotiation issues increases.

### Performance evaluations among different negotiation pairs

**Fig. 6:**
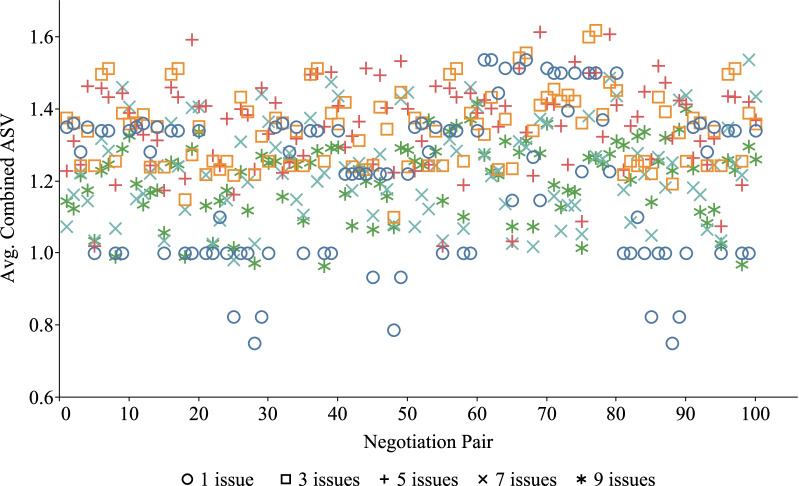
The combined ASV for different negotiation pairs on different issues

**Fig. 7:**
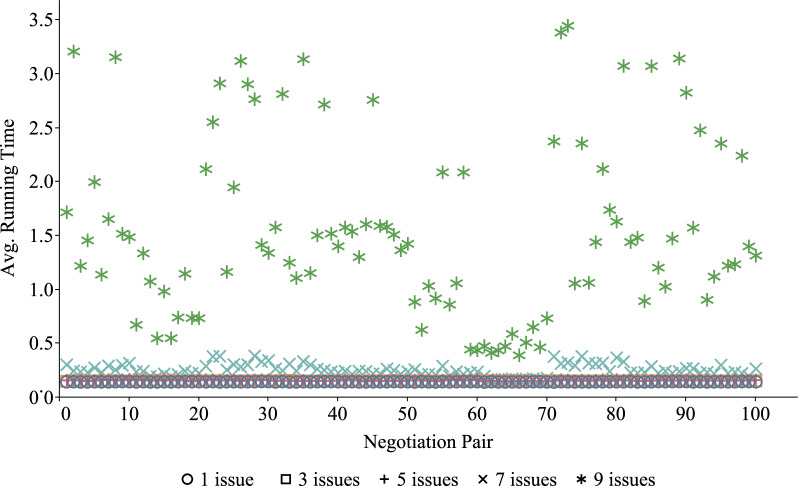
The running time for different negotiation pairs on different issues

**Fig. 8:**
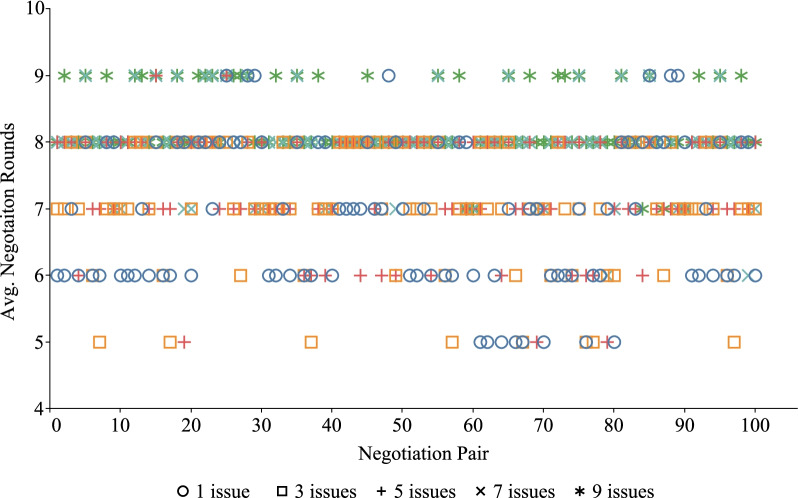
The negotiation rounds for different negotiation pairs on different issues

For different doctor and patient, the processes and results of PN-SDM may be different. Because their understanding of the same disease is different, their preference for the same negotiation issues may be different. However, our proposed negotiation is dynamic to a certain extent and can flexibly adapt to the real situation. To further evaluate the performance of the FCAN model, we simulated 10 doctor and 10 patient as experimental subjects and randomly set their preferences to compare the combined ASV, running time and negotiation rounds. There are 100 pairs of negotiations after permutation because each negotiation included one doctor and one patient. In the negotiation process, both sides used the win-win strategy to negotiate. The final results are shown in Figs. 6, 7, and 8.

Figure 6 shows the average combined ASV distribution of different negotiation pairs under different issues. As we can see from this figure, the distribution of the combined ASV on 1 to 9 issues is scattered. Specifically, the combined ASV obtained by DA and PA negotiation is not fixed and the value fluctuates between 0-2 regardless of the number of negotiation issues. This is because when the number of negotiation issues increases, the satisfaction of the DA and PA with the new negotiation issues is not fixed, which will directly affect the final combined ASV.

Figures 7 and 8 show the distribution of average running time and average negotiation rounds of different negotiation pairs under different issues, respectively. It can be seen from these two figures that the distribution of running time and negotiation rounds is relatively concentrated on different issues. In addition, when the number of issues increases from 1 to 9, the running time and negotiation rounds required for negotiation increase accordingly. The distribution of running time and negotiation rounds increases with the increase in the number of negotiation issues.

**Fig. 9:**
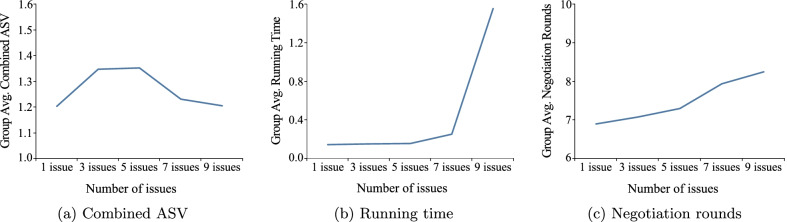
The group’s avg. combined ASV, running time, and negotiation rounds on different issues

Furthermore, the results of Fig. 9 are consistent with the conclusions above. Figure 9a–c shows the average combined ASV obtained by 100 groups of negotiation pairs (that is, 100 groups of DA and PA) and the average running time and negotiation rounds required for negotiation under different numbers of negotiation issues. It can clearly be observed that as the number of negotiation issues increases, more running time and negotiation rounds will be needed, but the combined ASV obtained will not be fixed.

The results of Figs. 6, 7, 8 and 9 all show that our negotiation model can effectively address the negotiation pressure caused by the increase in issues. More specifically, our model can effectively simulate negotiations between different doctor and patient and obtain satisfactory negotiation results in a limited amount of time. In these figures, the average combined ASV obtained by 100 pairs of DA and PA negotiation is greater than 1.0, while the running time required for negotiation is less than 4 s and the number of rounds required for negotiation is no more than 15.

## Conclusions

In this paper, we proposed a fuzzy constraint-directed agent-based multi-issue negotiation and recommendation framework to solve the bilateral PN-SDM problems. Compared with the artificial PN-SDM, the agent-based PN-SDM can complete multiple preferences negotiation in a few seconds. More importantly, our method can achieve satisfactory negotiation results in fewer negotiation rounds. The experimental results demonstrate that the presented FCAN model can flexibly deal with complex and real-world clinical situations through different strategies. It can usefully reduce the influence of multiple issues, time-space, and individual emotions to improve preferences negotiation efficiency. In addition, the proposed recommendation model can translate the negotiation results into treatment plans that achieve the purpose of treatment plan recommendations. In summary, this framework helps to realize PN-SDM intelligence and quantify preference information. It reduces the probability of high cost, time consumption, delayed response and decision fatigue. Relatedly, the method helps to improve the efficiency of negotiation in SDM and promote the clinical application of SDM with consideration of the value preferences of doctors and patients.

Although the proposed model is helpful for the completion of the PN-SDM process, it can still be further improved. In future work, the negotiation model will be improved by additional information sharing and the recommendation model will be enriched by more treatment data. In addition, the model proposed in this paper can be extended to more complex PN-SDM scenarios, such as a multi-agent system with an agent coalition. Moreover, the negotiation model and recommendation model will be tested by real doctors and patients in clinical practice. For example, we can also specifically study whether this method can indirectly improve patient compliance, improve the treatment effectiveness of diseases, or solve PN-SDM problems corresponding to other diseases.

## Data Availability

All data generated or analysed during this study are included in this published article.

## Abbreviations

SDM: Shared decision-making
PN-SDM: Preferences negotiation in shared decision-making
FCAN: Fuzzy constraint-directed agent-based negotiation
DFCSPs: Distributed fuzzy constraint satisfaction problems
DA: Doctor agent
PA: Patient agent
ASV: Aggregated satisfaction value
